# Quantitative analysis of tumor shrinkage due to chemotherapy and its implication for radiation treatment planning in limited-stage small-cell lung cancer

**DOI:** 10.1186/1748-717X-8-216

**Published:** 2013-09-16

**Authors:** Bing Xia, Jia-Zhou Wang, Qi Liu, Jing-Yi Cheng, Zheng-Fei Zhu, Xiao-Long Fu

**Affiliations:** 1 Department of Radiation Oncology, Shanghai Cancer Center, Fudan University, 270 Dong An Road, Shanghai 200032, China; 2 Department of Oncology, Shanghai Medical College, Fudan University, Shanghai, China; 3 Department of Radiation Oncology, Hangzhou Cancer Hospital, Hangzhou, China; 4 Department of Nuclear Medicine, Shanghai Cancer Center, Fudan University, Shanghai, China

**Keywords:** Small-cell lung cancer, Chemotherapy, Radiation therapy, Treatment planning

## Abstract

**Background:**

The optimal timing of chemoradiotherapy in limited-stage small-cell lung cancer (LS-SCLC) hasn’t been established, although evidence from studies supported that patients can benefit from early radiation therapy. The purpose of this study was to quantify tumor shrinkage in response to induction chemotherapy (IC), evaluate the impact of tumor shrinkage on radiation dosimetric parameters and determine its implication for the timing of radiation therapy for patients with LS-SCLC.

**Methods:**

Twenty patients with LS-SCLC who were treated with IC followed by concomitant radiation therapy were investigated retrospectively. Ten patients received 1 cycle of IC, and 10 patients received 2 cycles of IC. Pre-IC CT imaging was coregistered with a simulation CT, and virtual radiation plans were created for pre- and post-IC thoracic disease in each case. The changes in the gross target volume (GTV), planning target volume (PTV) and dosimetric factors associated with the lungs, esophagus and heart were analyzed.

**Results:**

The mean GTV and PTV for all of the patients decreased by 60.9% and 40.2%, respectively, which resulted in a significant reduction in the radiation exposure to the lungs, esophagus and heart. Changes in the PTV and radiation exposure of normal tissue were not significantly affected by the number of chemotherapy cycles delivered, although patients who received 2 cycles of IC had a greater decrease in GTV than those who received only 1 cycle of IC (69.6% vs. 52.1%, p = 0.273).

**Conclusions:**

Our data showed that targeting the tumor post-IC may reduce the radiation dose to normal tissue in patients with LS-SCLC. However, the benefit to the normal tissue was not increased by an additional cycle of IC. These findings suggest that the first cycle of chemotherapy is very important for tumor shrinkage and that initiating thoracic radiation therapy at the second cycle of chemotherapy may be a reasonable strategy for timing of radiation therapy in LS-SCLC treatment.

## Background

Small cell lung cancer (SCLC) is considered to be an aggressive form of lung cancer and is characterized by its good response to both chemotherapy and radiotherapy. The current standard of care for limited-stage SCLC (LS-SCLC) is thoracic radiation therapy (TRT) with concurrent chemotherapy [1]. In spite of a modest outcome improvement in recent decades, the overall survival remains poor. Furthermore, there are several controversies in the field, including how to optimize the TRT dose/fraction, the radiation target and the timing of chemoradiotherapy [2,3].

Although the optimum timing for TRT has not been established, previous studies support the notion that patients can benefit from early initiation of TRT and concomitant platinum-based chemotherapy [4,5]. However, the definition of “early” varies among these analyses (1–8 weeks) [6-9]. In recent clinical trials and in routine practice, concurrent TRT is frequently initiated during the first, second or third cycle of chemotherapy [10-14]. Undoubtedly, the first cycle of chemotherapy is the earliest time for delivering TRT. However, this is often accompanied by a high rate of treatment-related toxicities due to the relative extent of disease in SCLC [1,5,15], which potentially delayed subsequent chemotherapy and compromised the dose of chemotherapy, leading to prolonged overall treatment time. For patients who received TRT concurrently in the first cycle of chemotherapy, the proportion of patients completing planned chemotherapy was reported to be lower than 69% [5].

Because SCLC is sensitive to chemotherapy, it is logical that tumor shrinkage after induction chemotherapy (IC) can greatly influence radiation planning, thus reducing the radiation dose to critical normal organs. However, we know little about the pace of the SCLC volumetric response to IC or its impact on radiation planning for IC cycles. We hypothesized that a reduction of the tumor volume would have a significant influence on the radiation exposure of normal tissue after the first cycle of IC; however, this synergetic relationship would not be improved by subsequent cycles of IC. The purpose of this study was to quantify the magnitude of tumor shrinkage in LS-SCLC after one cycle of IC versus 2 cycles of IC. We also sought to evaluate its impact on radiation planning and its implication for timing of radiation therapy.

## Methods

### Patients

This retrospective study was approved by the Research and Ethics Committee of Fudan University Shanghai Cancer Center. To include patients who received 2 cycles of IC in this analysis, we searched our database from January 2006. The eligible patients had pathologic conformation of LS-SCLC received treatment with curative intent. We consecutively enrolled 10 patients with 1 cycle of IC and 10 patients with 2 cycles of IC. There were 16 males and 4 females. The median age was 59 years (45–66 years). Table 1 shows the site of the primary tumor and the positive lymph node station(s) for each patient. Generally, the staging workup included a complete blood count and liver function; bone scan; brain scan by computed tomography (CT) or magnetic resonance imaging; CT scan of the chest; and CT or ultrasound imaging of the abdomen. In addition, 16 patients underwent an ^18^ F-deoxyglucose (FDG) positron emission tomography/computed tomography (PET/CT) scan at the time of initial evaluation; of which, 9 and 7 patients were received 1 and 2 cycles of IC, respectively. Written informed consent was obtained from the patient for the publication of this report and any accompanying images.

**Table 1:** Patient characteristics

**No. of patients**	**Cycles of induction chemotherapy**	**Site of primary tumor**	Positive lymph node station*	**Stage**
1	1	Left lower lobe	4 L,4R, 10 L	T3N3
2	1	Right middle lobe	2R, 4R, 10R	T2N2
3	1	Right upper lobe	3P, 4 L, 4R, 7, 10R	T3N3
4	1	Right lower lobe	4R, 7, 8, 10R	T3N2
5	1	Left upper lobe	2 L, 3A, 4 L, 5, 7, 10 L	T2N2
6	1	Right lower lobe	7, 10R	T3N2
7	1	Right upper lobe	1, 2R, 4 L, 4R, 7, 10R	T3N3
8	1	Left upper lobe	4 L, 5, 10 L	T2N2
9	1	Left hilus	2 L, 2R, 3A, 4 L, 4R, 5, 6, 7, 8, 10 L	T3N3
10	1	Left upper lobe	3A, 4 L, 4R, 5, 6, 7, 10 L	T2N3
11	2	Right lower lobe	4R, 7, 10R	T2N2
12	2	Right upper lobe	2R, 4R, 7, 10R	T4N2
13	2	Left upper lobe	4 L, 6, 7, 10 L	T3N2
14	2	Left upper lobe	4 L, 4R, 5, 6, 10 L	T4N3
15	2	Right hilus	4R, 10R	T3N2
16	2	Left upper lobe	2 L, 4 L, 4R, 5, 6, 7, 10 L	T3N3
17	2	Left lower lobe	2R, 4R, 4 L, 6, 7, 8, 10 L	T3N3
18	2	Left upper lobe	1, 2 L, 3A, 6, 10 L	T2N3
19	2	Right hilus	3A, 4R, 10R	T3N2
20	2	Left upper lobe	2R, 4 L, 5, 6, 10 L	T3N3

*According to the definition for lymph node stations in International Association for the Study of Lung Cancer Map.

### Treatment

All of the patients in this study were treated with concomitant chemoradiotherapy. Chemotherapy was administered as a combined etoposide/cisplatin regimen, with doses of 70 mg/m^2^ for etoposide from day 1 to day 4 and 25 mg/m^2^ for cisplatin from day 1 to day 3 administered intravenously at an interval of 3 weeks.

### Image registration

All of the pre-treatment CT imaging was obtained within 1 week before treatment initiation. For the 16 patients with staging PET/CT scans, the imaging data sets (including both the PET and CT scans) were transferred to a radiotherapy planning workstation (Pinnacle 7.6 TPS, Philips Medical Systems, Andover, MA). For the remaining 4 patients, diagnostic chest CT scans with contrast enhancement were used. The simulated contrast-enhanced CT scans performed for radiotherapy planning were coregistered with the pre-treatment CT imaging using the image fusion tools available in the Pinnacle software. The accuracy of this process was confirmed visually. When necessary, the imaging was manually adjusted to improve the matching of immobile anatomic landmarks close to the tumor. No breath control was used during image acquisition.

### Target volume delineation

All of the targets were contoured by a single experienced investigator to limit inter-user variability, using the lung windows to define the intrapulmonary component of the tumor and the soft tissue windows to delineate the mediastinal component and the associated lymph nodes. In each case, the pre-IC gross target volume (GTV) included the primary lung tumor and the involved mediastinal lymph node regions in a single contoured structure. The post-IC GTV included the residual primary tumor and all of the clinical and radiological involved lymphatic regions. When the enlarged lymph nodes disappeared after chemotherapy, the previously involved lymph node regions were still included in the radiation target by reviewing the pre-IC CT scan. Elective treatment of clinically uninvolved lymphatic regions was not carried out. The planning target volume (PTV) was formed with a margin of 1–1.5 cm according to the position of the target volume. No specific clinical target volume (CTV) was used in this population, considered that a relatively large margin of 1.5 cm was commonly used in most of the patients and that part of the CTV has been included besides setup error and target motion [16].

If the edge of a tumor mass could not be defined with CT imaging because of a lack of contrast between the mass and the atelectatic lung or mediastinal structures, a PET component was used to derive the best estimate of the tumor edge when a PET/CT was available. Enlarged lymph nodes without FDG uptake were considered tumor negative, whereas small (short axis <1 cm) lymph nodes with visible FDG uptake were considered tumor positive.

Contouring of the lung was performed automatically by the treatment planning system. For the calculation of the V5, the V20 (the percentage of lung receiving more than 5 and 20 Gy) and the mean lung dose (MLD), the volume of both lungs minus the GTV was used. The esophagus was delineated from just below the larynx to the gastro-esophageal junction. The spinal cord was drawn throughout the whole CT scan and was considered to be located at the inner margin of the bony spinal canal. The heart was contoured from its caudal part at the apex until its cranial level at the beginning of the large vessels.

### Radiation treatment planning and dosimetry

A virtual IMRT (intensity modulated radiotherapy) plan was created for the pre- and post-IC PTV in each case using the Pinnacle planning system. This virtual IMRT generally consisted of five to eight fields using 6-MV photons. The planned radiation dose was 60 Gy. The selected dose was 60 Gy instead of 45 Gy because of the nature of virtual planning in this study and the trend of using a high dose in LS-SCLC treatment [17]. All of the plans aimed to achieve the objectives of the International Commission on Radiation Units and Measurements by conforming the 95% isodose volume (at a minimum) as tightly as possible to the PTV, while respecting the maximal dose constraints to the spinal cord (45 Gy). As per the criteria, radiation doses to other normal tissues at risk were minimized (giving priority to the lungs, heart and esophagus). We took into consideration both the conformity and the homogeneity of PTV, but the former was given top priority.

For the lung, the V5, the V20 and the MLD were analyzed as predictors for radiation pneumonitis. Because no consistent dosimetric parameters are known to predict early and late esophageal toxicity, the V40, the V50, the V60, the maximal dose, the mean dose to the esophagus and the absolute esophageal volume which received a radiation dose > 60 Gy were analyzed. The mean dose to the heart, as well as the maximal dose and the V30, were also recorded.

### Comparative analysis

The results were expressed as the mean and standard deviation of the studied indices. The differences between the GTV, PTV and doses to the organ at risk, based on pre- and post-IC planning, were compared for each case using a paired *t*-test. The subgroup comparison between the 10 patients who received 1 cycle of IC (group 1) and the 10 patients who received 2 cycles of IC (group 2) was performed using the relative change (RC). The RC was calculated using the formula (*X*2-X1)/X1*100%, here, X1 and *X*2 were the variants pre- and post-IC for GTV, PTV and the parameters for evaluating normal tissue toxicities, respectively. A rank-sum test was performed to evaluate the differences between the two subgroups. P-values less than 0.05 were considered significant. A software package SPSS 13.0 (IBM, Somers, New York) was used for statistical analysis.

## Results

### Radiotherapy plans

In some cases, the PTV was very close to the spinal cord, then the PTV was modified manually to meet the dose constrain of spinal cord for clinical purposes. The V_PTV60_ (percentage of PTV receiving more than 60 Gy) in the pre- and post-IC planning was 95.1 ± 0.4% (range, 94.5-96.0%) and 95.2 ± 0.5% (range, 94.5-96.0%), respectively. The maximal dose to the spinal cord was 44.3 ± 0.6 Gy (range, 43.2-45.3 Gy) and 43.9 ± 1.3 Gy (range, 40.3-45.9 Gy), respectively, in the pre- and post-IC planning.

### Changes of GTV and PTV

**Figure 1:**
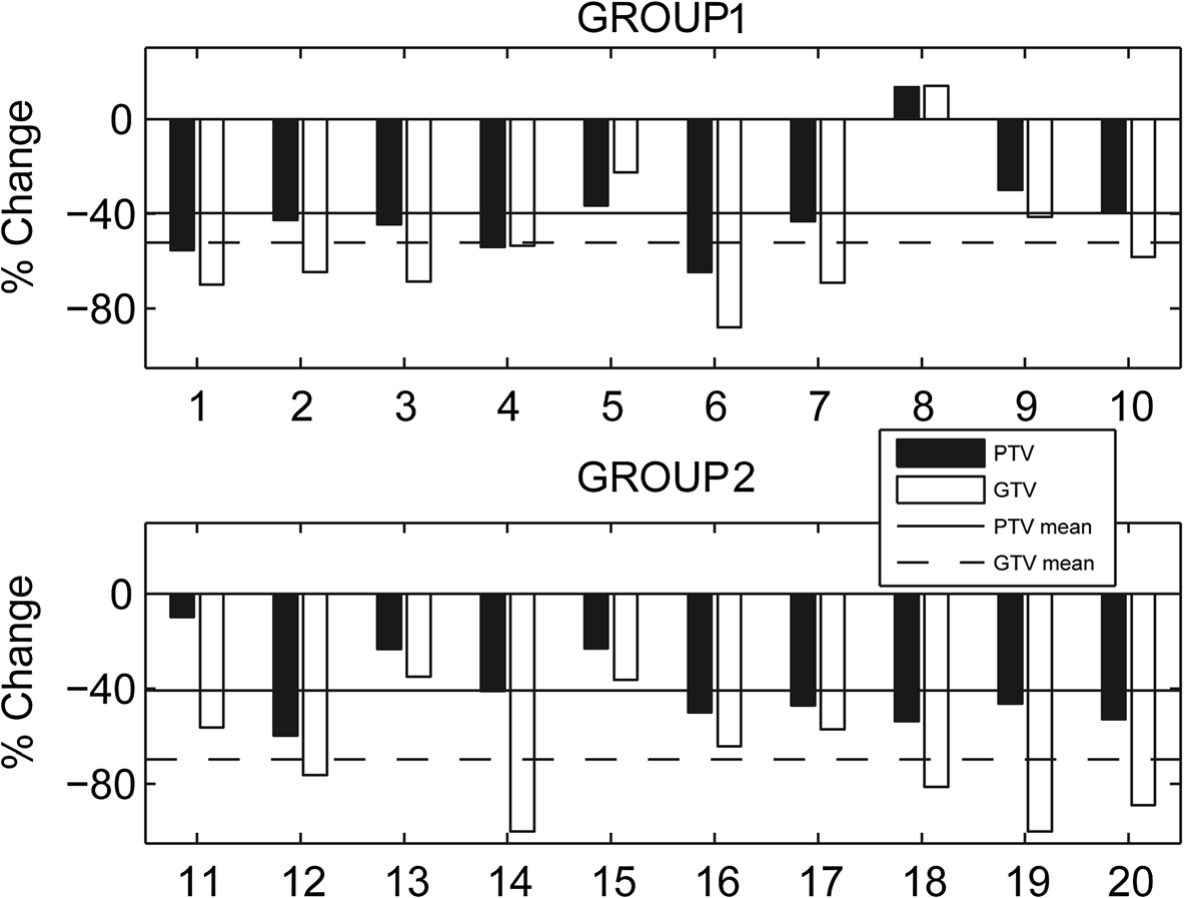
**Relative reduction in gross target volume (GTV) and planning target volume (PTV) after induction chemotherapy in group1 (n = 10) and in group 2 (n = 10).** For the 2 patients with a complete response, the changes of GTV were recorded as 100%.

The majority of the patients (95%) responded well to IC (2 complete responses, 17 partial responses and 1 stable disease). Table 2 shows the pre- and post-IC volumes of GTV and PTV. For all of the patients, the mean decreases in GTV and PTV were 60.9% and 40.2%, respectively. The mean decrease in PTV was similar between the 2 subgroups (39.6% vs. 40.8%, p = 0.97), although the degree of GTV decrease in group 2 was higher than in group 1 (69.6% vs. 52.1%, p = 0.273; Figure 1).

**Table 2:** Gross target volume and planning target volume in 20 patients with LS-SCLC

	**Gross target volume (cm**^**3**^**)**		**Planning target volume (cm**^**3**^**)**	
	**Pre-chemotherapy**	**Post-chemotherapy**	**Pre-chemotherapy**	**Post-chemotherapy**
Group1	233 ± 167	102 ± 75	712 ± 336	422 ± 220
Group2	245 ± 217	81 ± 56	731 ± 380	397 ± 128
All patients	240 ± 184	92 ± 64	722 ± 341	410 ± 171

Patients in group 1 (n = 10) received 1 cycle of induction chemotherapy and patients in group 2 (n = 10) received 2 cycles. There was no significant difference in the distribution of gross target volume and planning target volume pre-treatment between the two groups; p-values were 0.910 and 0.850, respectively.

### Normal tissue radiation exposure

**Figure 2:**
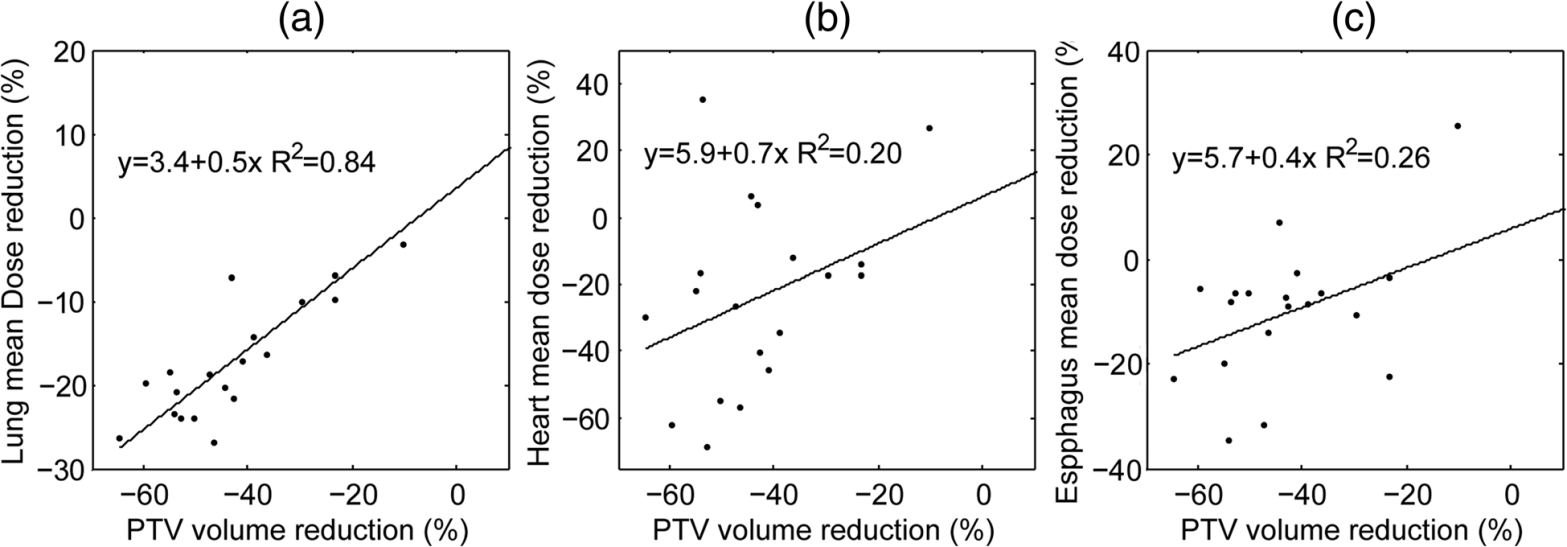
**Correlations between decreases in the mean dose to the lung, esophagus and heart and reduction of the planning target volume (PTV) in 20 patients with LS-SCLC who received induction chemotherapy.** The **a**, **b** and **c** correspond to the lung, esophagus and heart, respectively.

An overview of the radiation exposure of normal tissues that compares the pre- and post-IC plans is shown in Table 3. The tumor shrinkage caused by IC translated into significant changes in most of the dosimetric factors analyzed. Figure 2 shows correlations between decreases in the mean dose to the lung, esophagus as well as heart, and shrinkage in the PTV. Subgroup comparisons between patients receiving 1 or 2 cycles of IC showed that there were no significant differences for any of these variations (Table 4).

**Table 3:** Comparison of normal tissue radiation exposure pre- and post-induction chemotherapy

		**Pre-chemo plan**	**Post-chemo plan**	**p-value**
*Lung*				
	V_5_ (%)	59.0 ± 6.4	54.8 ± 6.0	0.000
	V_20_ (%)	32.3 ± 5.1	28.2 ± 4.0	0.000
	MLD (Gy)	18.6 ± 2.3	15.5 ± 1.9	0.000
*Heart*				
	V_30_ (%)	24.2 ± 21.0	17.1 ± 17.0	0.001
	Mean (Gy)	18.0 ± 11.0	13.7 ± 9.0	0.000
	Max (Gy)	64.3 ± 10.9	61.5 ± 10.1	0.263
*Esophagus*				
	V_40_ (%)	45.4 ± 20.0	39.6 ± 16.6	0.023
	V_50_ (%)	39.1 ± 22.8	33.1 ± 17.8	0.038
	V_60_ (%)	27.6 ± 21.1	20.2 ± 14.3	0.022
	AV_60_ (cm^3^)	8.7 ± 5.9	6.7 ± 5.0	0.012
	Mean (Gy)	31.9 ± 10.6	28.4 ± 8.7	0.004
	Max (Gy)	65.2 ± 3.0	65.0 ± 2.7	0.688

*Abbreviations:AV*_60_ Absolute volume > 60Gy.

**Table 4:** Comparison of the decrease magnitude between the 2 subgroups

		**Group1**	**Group2**	**P-value**
*Lung*				
	V5 (%)	−4.8 ± 8.0	−9.1 ± 4.5	0.212
	V20 (%)	−12.3 ± 13.3	−11.6 ± 6.8	0.473
	MLD (%)	−14.6 ± 11.4	−17.2 ± 7.9	0.678
*Heart*				
	V30 (%)	−16.5 ± 36.4	−34.4 ± 53.4	0.212
	Mean (%)	−15.8 ± 17.0	−28.4 ± 36.5	0.212
	Max (%)	−2.3 ± 1.9	−10.5 ± 18.1	0.473
*Esophagus*				
	V40 (%)	−10.8 ± 17.2	−9.7 ± 17.8	0.85
	V50 (%)	−14.8 ± 21.1	−10.2 ± 22.6	0.385
	V60 (%)	−28.0 ± 48.9	−21.9 ± 24.8	0.791
	AV_60_ (%)	−28.0 ± 48.9	−21.9 ± 24.8	0.791
	Mean (%)	−11.0 ± 12.3	−7.6 ± 14.8	0.385
	Max (%)	−0.6 ± 4.7	0.1 ± 3.3	0.734

*Abbreviations*: *AV*_60_ Absolute volume > 60 Gy.

Patients in group 1 (n = 10) received 1 cycle of induction chemotherapy and patients in group 2 (n = 10) received 2 cycles.

## Discussion

Our study showed that there was a significant decrease in the GTV (61%) and PTV (40%) after IC in LS-SCLC. This decrease translated into a remarkable decrease in the radiation exposure of normal tissue, including the lung, esophagus and heart. Compared with the modest decreases caused by IC in non-small-cell lung cancer (37% in GTV and 26% in PTV) [18], this benefit was more obvious in SCLC. However, we did not find a significant difference in the degree of reduction in PTV or radiation dose to the normal tissue between patients who received 1 cycle of IC and those who received 2 cycles of IC; although patients who received 2 cycles of IC had a greater decrease in GTV compared with those who received only 1 cycle of IC. These findings indicated that administrating TRT at the second cycle of chemotherapy is a reasonable time for LS-SCLC. Doing so could balance the conflict between delivering early TRT and decreasing treatment-related toxicity.

The optimal timing for TRT in LS-SCLC is debatable. Over the past two decades, many clinical trials examining the effect of early or late TRT on survival in LS-SCLC have been conducted, and the results have varied. De Ruysscher et al. pooled 7 studies investigating the timing of TRT in LS-SCLC and found no significant difference between early or late TRT. When only trials that used platinum chemotherapy concurrent with TRT were considered, a significantly higher 5-year survival was observed when TRT was initiated within 30 days after the start of chemotherapy [6]. Another meta-analysis conducted by Spiro et al. examined the proportion of patients who completed chemotherapy in 8 studies and compared the effects of early and late TRT. The researchers found that the proportion of patients completing all cycles of chemotherapy was noticeably less in the early versus late TRT arm in 5 of 8 trials. Only 3 trials had a similar proportion of patients who received their intended chemotherapy in the early and late TRT arms, and survival benefits of early TRT were observed exclusively in these 3 trials [5]. These striking findings suggest that it is essential to ensure that the delivery of chemotherapy is optimal when administered with early TRT. Often, at the time of SCLC diagnosis, the disease appears as a conglomerate central mass involving the parenchyma and mediastinal lymph nodes [3]. If TRT is initiated on the first day of the treatment, this constitutes a relatively large radiation field to safely cover the tumor. It is challenging for radiation oncologists to plan treatment without compromising the chemotherapy intensity and the therapy duration.

A benefit of tumor shrinkage with IC is that the radiation fields can be made smaller. Therefore, the dose received by the surrounding structures is decreased, which potentially leads to less normal tissue toxicity and could contribute to a full completion of each modality. In our study, the majority of the patients responded well to IC, which produced an average 60.9% decrease of GTV and a 40.2% decrease of PTV in LS-SCLC, resulting in a significant decrease in the radiation dose to the lungs, heart and esophagus. Thus, delivering TRT after IC in LS-SCLC provides an opportunity to reduce the TRT-related toxicity and might be helpful for completion of the intended chemotherapy.

In this study, involved-field irradiation was used in designing the target volume. For primary tumor delineation in post-IC treatment planning, only the post-IC volume was contoured, which was based on the evidence that the main local failure developed inside the TRT field [16,19,20]. Parenchyma recurrence outside of the TRT field is very rare, possibly because the area occupied by the pretreatment tumor mass is filled by normal lung tissue after chemotherapy, which makes it safe to irradiate only the residual tumor. However, it is risky to irradiate only the enlarged mediastinal lymph nodes shown in post-IC CT imaging. Although enlarged lymph nodes were no longer visible in the post-IC CT imaging, there may have been residual tumor cells in the mediastinal lymph region because it is very hard to eliminate a solid tumor by chemotherapy alone. Therefore, for planning of the lymph nodes, the pretreatment anatomic sites of the involved zones in the baseline CT scan have been delineated in many reports [10,14,21]. In addition, elective node irradiation was not allowed in our study because the safety of this approach has been questioned [22,23]. Recently, Van Loon et al. reported a low isolated node failure rate (3%) in LS-SCLC when only the involved mediastinal lymph nodes were irradiated based on the pretreatment PET scan [14,24]. A similar result was also shown in the Shirvani study [21]. These findings support the decision to abandon elective nodal irradiation in light of the excellent performance of contemporary functional imaging.

In our study, the relative decrease in GTV was high than that of PTV, primarily because we used the target contouring method described above. SCLC often presented with lymph node metastasis at diagnosis, and the primary tumor and mediastinal involved zones were always included in the field. Thus, the contouring method weakened the contribution of the primary tumor shrinkage to the sparing of normal tissue. Similarly, this can also be used to explain why there was no significant difference in the radiation dose to normal tissue for patients receiving 1 or 2 cycles of IC. Although the reduction of GTV was nearly 70% in group 2 and 50% in group 1, similar PTV reductions of 40% were observed in both groups. Moreover, the space between the primary tumor and the mediastinal lymph station did not change due to the administration of IC. In addition, PTV also included a 1.5 cm margin around the GTV, making the relative reduction in PTV following IC smaller than the relative reduction in GTV.

Although SCLC is considered to be a radiosensitive disease, moderate doses of TRT (45–54 Gy) have been associated with a high frequency of local failure (30-50%), suggesting that more aggressive TRT may offer additional therapeutic advantages [1,25]. It can be expected that targeting the tumor volume after IC will benefit TRT dose escalation or intensification. Currently, two ongoing randomized Phase III trials are exploring high dose radiation in LS-SCLC. The first trial (CALGB 30610/RTOG 0538) initiated TRT on the first day of the first cycle of chemotherapy. The other trial (CONVERT, running in both Europe and Canada) administered TRT on the first day of the second cycle of chemotherapy. The completion of the two trials will provide more data about the differences in toxicities and survival outcomes when delivering TRT concurrently with the first or second cycle of chemotherapy.

There are several limitations to this analysis, including those inherent to retrospective studies. In the present study, we consecutively enrolled 20 patients. There was no imbalance in the distribution of patients and tumor characteristics, including tumor volume and the number of patients receiving a PET scan between the two subgroups. An improved approach to answer the questions raised in this study would be to prospectively conduct a self-paired comparison based on the individual images obtained at each cycle of IC. In addition, we used dosimetric parameters as a surrogate to evaluate the normal tissue toxicity in this study. Thus, the benefits from IC are only theoretical clinical gains because of many largely unknown patient and chemotherapy factors that may influence the toxicity of radiotherapy; however, these unknown factors should be resolved in the setting of prospective clinical studies.

## Conclusions

In summary, our findings showed that targeting the tumor post-IC may reduce the radiation dose to normal tissue in patients with LS-SCLC. However, the benefit to normal tissue was not increased by an additional cycle of IC. These findings suggest that the first cycle of chemotherapy is very important for tumor shrinkage, and that initiating TRT at the second cycle of chemotherapy may be a reasonable strategy for timing of radiation therapy in the treatment of LS-SCLC.

## Competing interests

The authors declare that they have no competing interests.

## Authors’ contributions

BX and XLF designed this study, performed much of the work, and drafted the manuscript. JZW performed the treatment plans and imaging fusion. QL collected clinical data. JYC interpreted PET and CT scan results. ZFZ participated in the analysis and the data interpretation. All authors read and approved the final manuscript.
